# A LONGITUDINAL INQUIRY OF PATIENT-PROVIDER RELATIONSHIPS AMONG AN UNDERSTUDIED GERIATRIC POPULATION

**DOI:** 10.1093/geroni/igac059.1857

**Published:** 2022-12-20

**Authors:** Rosana Bravo, Angela Gutierrez

**Affiliations:** Western University of Health Sciences, Pomona, California, United States; Ohio University, Heritage College of Osteopathic Medicine, Athens, Ohio, United States

## Abstract

Patient-provider relationships have a direct impact on patient outcomes. This study explored patient-provider relationships among an understudied geriatric population of color— foreign-born Latinos¬ participating in an all-inclusive specialized program aimed at controlling patients’ costs and enhancing access to care. Thirteen older adult Latinos with multimorbidities from nine Program of All-Inclusive Care for the Elderly (PACE) centers in Southern California were recruited. Researchers conducted three in-depth interviews in Spanish with each participant (39 interviews total) over 13 months. The first interviews were conducted face-to-face and lasted one hour on average. Subsequent interviews were conducted over the phone (Range: 60-90 minutes). Data were analyzed using codes, identifying categories and themes. The concepts of time and trust were used to analyze the process of relationship development and to capture changes over time. The patient-provider relationship developed on a continuum across time and trust, establishing three stages to the patient-provider relationship. In the first stage emerged the concept of el buen doctor (the good doctor). In the second stage, trust was perceived to have been established, and was only strengthened as the doctor continued to demonstrate trustworthy characteristics over time. The third stage embodied all that a person of trust was plus an additional advocacy dimension. The longitudinal and specialized geriatric program design illuminated the nature of quality of care and patients’ perceptions on relationship development over time. Controlling patient/provider costs and enhancing access to care in an all-inclusive program are beneficial in enhancing patient-provider relationships.

